# Effect of ethanol supplementation on the transcriptional landscape of bionanocellulose producer *Komagataeibacter xylinus* E25

**DOI:** 10.1007/s00253-019-09904-x

**Published:** 2019-06-06

**Authors:** Małgorzata Ryngajłło, Paulina Jacek, Izabela Cielecka, Halina Kalinowska, Stanisław Bielecki

**Affiliations:** Institute of Technical Biochemistry, Lodz University of Technology, B. Stefanowskiego 4/10, 90-924 Lodz, Poland

**Keywords:** *Komagataeibacter*, Bacterial cellulose, Bionanocellulose, Ethanol, RNA-seq, Transcriptomics

## Abstract

Ethanol exerts a strong positive effect on the cellulose yields from the widely exploited microbial producers of the *Komagataeibacter* genus. Ethanol is postulated to provide an alternative energy source, enabling effective use of glucose for cellulose biosynthesis rather than for energy acquisition. In this paper, we investigate the effect of ethanol supplementation on the global gene expression profile of *Komagataeibacter xylinus* E25 using RNA sequencing technology (RNA-seq). We demonstrate that when ethanol is present in the culture medium, glucose metabolism is directed towards cellulose production due to the induction of genes related to UDP-glucose formation and the repression of genes involved in glycolysis and acetan biosynthesis. Transcriptional changes in the pathways of cellulose biosynthesis and c-di-GMP metabolism are also described. The transcript level profiles suggest that Schramm-Hestrin medium supplemented with ethanol promotes bacterial growth by inducing protein biosynthesis and iron uptake. We observed downregulation of genes encoding transposases of the IS*110* family which may provide one line of evidence explaining the positive effect of ethanol supplementation on the genotypic stability of *K. xylinus* E25. The results of this study increase knowledge and understanding of the regulatory effects imposed by ethanol on cellulose biosynthesis, providing new opportunities for directed strain improvement, scaled-up bionanocellulose production, and wider industrial exploitation of the *Komagataeibacter* species as bacterial cellulose producers.

**Electronic supplementary material:**

The online version of this article (10.1007/s00253-019-09904-x) contains supplementary material, which is available to authorized users.

## Introduction

Bacterial cellulose (bionanocellulose, BNC) has a wide range of possible applications in various sectors of biotechnology, medicine, and industry due to its outstanding mechanical properties, high chemical purity (lack of lignin and hemicellulose), biocompatibility, and biodegradability (Cacicedo et al. 2016; Gama et al. 2016; Ludwicka et al. 2018). Another advantage of BNC for industrial applications is that its physical properties can be modified during cultivation, such as by the use of water-soluble and insoluble polymers (Cacicedo et al. 2016; Cielecka et al. 2018). Acetic acid bacteria of the *Komagataeibacter* genus have been shown to be exceptionally efficient bacterial cellulose producers (Lin et al. 2013; Valera et al. 2015). The recently accumulating genome sequences of this genus enable better understanding of the molecular mechanisms controlling cellulose synthesis and thus open possibilities for engineering strains to improve BNC production.

Cellulose is a water-insoluble exopolysaccharide composed of β-1,4-glucan chains. It has been speculated that BNC gives bacterial cells protection from UV radiation and desiccation (Williams and Cannon 1989). When growing statically in a liquid medium, the synthesis of a cellulose membrane enables the retention of cells close to the air-liquid interface, where they have access to nutrients from the medium and the concentration of oxygen is high (Williams and Cannon 1989; Czaja et al. 2007). The cellulose biosynthesis pathway and the general metabolism model of a *Komagataeibacter* sp. have been delineated (Velasco-Bedrán and López-Isunza 2007). Bacterial cellulose synthase (BCS) is the key enzyme which catalyzes the polymerization of UDP-glucose into cellulose. First discovered and studied in *Komagataeibacter xylinus*, BCS consists of four subunits (Wong et al. 1990; Umeda et al. 1999; Jedrzejczak-Krzepkowska et al. 2016). Subunits A and B (BcsA and BcsB) are responsible for β-glucan chain formation (Römling and Galperin 2015; Morgan et al. 2016). The role of the other two subunits (BcsC and BcsD), which influence the efficiency of cellulose synthesis, is still unclear (Saxena et al. 1994; Hu et al. 2010; Iyer et al. 2011; Du et al. 2016; Nojima et al. 2017). The genes that encode the subunits of BCS form an operon (*bcsABCD*) and are flanked by the genes encoding endo-β-1,4-glucanase (CMCAx), the cellulose complementing factor (CcpAx) and β-glucosidase (BglAx), which influence the process of cellulose synthesis (Jedrzejczak-Krzepkowska et al. 2016). Expression of the *bcs* operon has been studied extensively in *Salmonella typhimurium*. It was found to be constitutive, although it fluctuates depending on the growth phase and environmental conditions (Zogaj et al. 2001). The stimulation of cellulose biosynthesis occurs at a posttranslational level and depends on c-di-GMP (bis-(3′,5′)-cyclic di-guanosine-mono-phosphate), a universal bacterial second messenger, which activates BcsA allosterically by binding to its PilZ domain (Fujiwara et al. 2013; Römling et al. 2013). The c-di-GMP turnover in the cell is under the control of proteins with opposite enzymatic activities: diguanylate cyclases (DGCs) and c-di-GMP-specific phosphodiesterases (PDEs), which catalyze c-di-GMP formation and degradation, respectively (Tal et al. 1998; Römling et al. 2013). It has further been demonstrated that DGCs are characterized by the presence of a GGDEF domain, whereas PDEs contain an EAL domain (Ausmees et al. 2001; Simm et al. 2004). Three operons (*cdg1*, *cdg2*, *cdg3*), which encode homologous isoforms of DGC and PDE, have been discovered and studied in *K. xylinus* (Tal et al. 1998). The first operon (*cdg1*) was found to be responsible for 80% of both PDE and DGC activity, whereas *cdg2* and *cdg3* were responsible for 15% and 5%, respectively. The PDE1 (AxPDEA1) protein has been shown to function as an O_2_ sensor since oxygen binding to its heme-based Per-Arnt-Sim (PAS) domain results in EAL domain inactivation (Chang et al. 2001). The DGC2 (AxDGC2) protein contains a flavin cofactor-binding PAS domain, which influences DGC activity depending on fluctuations in the cellular redox status or oxygen concentration (Qi et al. 2009). Therefore, through the DGC and PDE sensors, oxygen availability and cellular redox status influence c-di-GMP levels and thus BNC biosynthesis. As has been shown recently by the authors of the present study, the genome of *K. xylinus* E25 contains 15 genes encoding GGDEF-EAL proteins, including the three canonical operons, which suggests that the c-di-GMP signaling network may be very extensive (Ryngajłło et al. 2018). In the same study, we showed that, apart from the cellulose, *K. xylinus* E25 may also synthesize acetan, a water-soluble exopolysaccharide (EPS). A cluster of 17 genes encoding enzymes involved in the synthesis of nucleotide sugar precursors of acetan (UDP-glucuronate, GDP-mannose, dTDP-rhamnose) was discovered and described, together with its export machinery. Importantly, the biosynthesis of this EPS competes for glucose with cellulose formation (Griffin et al. 1996; Kornmann et al. 2003).

One of the factors that hinder the wider commercial use of BNC is its price. Therefore, improved processes of cellulose production are sought, which would lower costs and allow for the development of new applications. Alternative substrates, such as ethanol, acetic acid, lactic acid, and sodium citrate have been tested for their ability to promote cellulose production (Matsuoka et al. 1996; Naritomi et al. 1998a, b; Li et al. 2012; Molina-Ramírez et al. 2018). Ethanol has been shown to have a particularly strong inducing effect on BNC synthesis in the case of the *Komagataeibacter* species (Naritomi et al. 1998b; Krystynowicz et al. 2002; Park et al. 2003; Dubey et al. 2017; Liu et al. 2018; Molina-Ramírez et al. 2018). Ethanol is naturally present in the environments from which many *Komagataeibacter* species are isolated, such as vinegar or the fermented tea beverage, Kombucha. In acetic acid bacteria (AAB), ethanol undergoes partial oxidation in the periplasm by membrane-bound alcohol dehydrogenase (ADH) and aldehyde dehydrogenase (ALDH) (Mamlouk and Gullo 2013; Adachi and Yakushi 2016). These reactions are coupled to oxygen reduction by quinol oxidases through ubiquinone (Arai et al. 2016). Thus, oxidation of ethanol to acetate in the periplasm generates a proton motive force that can be used for ATP synthesis or other bioenergetic work. Ethanol can also be completely oxidized in the cytosol by cytosolic ADH and ALDH. Moreover, some AAB, including *Acetobacter* and *Komagataeibacter*, when grown on ethanol, temporarily accumulate acetate until ethanol is consumed (Yunoki et al. 2004; Sakurai et al. 2011; Mamlouk and Gullo 2013). The acetate is then completely oxidized in the cytosol via the tricarboxylic acid (TCA) cycle or by acetyl CoA synthase (ACS), and can therefore be utilized as a source of both carbon and energy.

Why ethanol supplementation contributes to higher BNC yields is not yet fully understood. In one study, it was suggested that ethanol results in higher ATP levels, which in turn have an inhibitory effect on the activity of glucose-6-phosphate dehydrogenase, thereby attenuating glucose flow into the pentose phosphate pathway (PPP) (Naritomi et al. 1998b). In another study, the activity of glucokinase (hexokinase in the original work) was found to be induced in the presence of ethanol (Li et al. 2012). The conclusion that the authors of both studies draw is that ethanol functions as an energy source for ATP generation but is not utilized as a substrate for BNC biosynthesis. These postulates are supported by a study employing isotope-labeled carbon, in which it was observed that ethanol was not used in BNC biosynthesis as a substitute for glucose (Yunoki et al. 2004). Similarly, it was concluded that ethanol serves as an energy source, thereby improving the effective use of glucose for cellulose synthesis, by preventing its utilization for energy acquisition.

The effect of ethanol supplementation on gene expression has not been reported for the *Komagataeibacter* spp. In this paper, we compare the global transcriptional landscape of *K. xylinus* E25 growing in the medium supplemented with ethanol to that of a culture deprived of ethanol, which was used as a control. The purpose is to provide a broad overview of the cellular response to the presence of ethanol with a particular focus on its ability to improve BNC production.

## Material and methods

### Culture conditions and RNA sequencing

#### Bacterial strain and growth media

The bacterial strain used was *K. xylinus* E25 (Bowil Biotech Ltd., Władysławowo, Poland). Unless otherwise stated, this strain was cultured in SH medium (basal medium; (Hestrin and Schramm 1954)) at 30 °C under static conditions. One liter of the culture medium contained 20.0 g glucose (POCh, Gliwice, Poland), 5.0 g yeast extract (BTL, Łódź, Poland), 5.0 g bacterial peptone (BTL, Łódź, Poland), 2.7 g sodium phosphate dibasic (Chempur, Piekary Śląskie, Poland), 1.15 g citric acid (Chempur, Piekary Śląskie, Poland), and 0.5 g magnesium sulfate (Chempur, Piekary Śląskie, Poland). When necessary, the basal medium was supplemented with 1% EtOH (ethanol medium). The initial pH of the medium was adjusted to 5.7 with 80% acetic acid (Chempur, Piekary Śląskie, Poland).

#### Time course experiment

*K. xylinus* E25 was cultured in 10-mL test tubes, each containing 5 mL of SH medium (with or without the addition of EtOH) inoculated with a single colony. On each day, the cellulose membranes were collected from three replicated cultures of each condition. The BNC membranes were next treated with 2% solution of NaOH for one night and 1.5% acetic acid for 4 h. They were then carefully washed in distilled water until neutral pH was reached. The purified BNC membranes were then dried at 30 °C to a constant weight and weighed. Bacterial growth in the culture was assessed each day after cellulose membrane degradation, which commenced 24 h before the end of the culture. The membranes were degraded using 200 μL of Ultraflo Max cellulase (Novozyme, Bagsvaerd, Denmark) diluted 5:3 with SH medium. The culture was next diluted in series and 100 μL of the diluted culture was spread on the solid SH medium in three independent replicates. After 5 days of incubation, the colony-forming units (CFU) were counted. The concentration of glucose in the medium was assessed using a GLUCOSE enzymatic test (BioMaxima, Lublin, Poland). The gluconic acid concentration was determined as the sum of D-gluconic acid and D-gluconolactone using K-GATE enzymatic tests (Megazyme, Bray, Ireland). The concentrations of acetic acid and ethanol, respectively, were determined using K-ACET and K-ETOH enzymatic tests (Megazyme, Bray, Ireland). The intracellular ATP content was determined using a BacTiter-Glo™ Microbial Cell Viability Assay (Promega Inc., Madison, USA). All assays were conducted according to the manufacturers’ protocols. The results are presented in Fig. S1 and Table S1 of Online Resource 1.

#### RNA-seq experiment

For the RNA-seq experiment, the *K. xylinus* E25 strain was cultured in 10-mL test tubes containing 5 mL of a culture medium at 30 °C under static conditions for 4 days. In total, 6 cultures were prepared, 3 with SH medium and 3 with SH medium containing 1% ethanol. To the culture was added a 1% of cellulase solution (v/v) (from *Trichoderma reesei* ATCC 26921, Sigma-Aldrich, Steinheim, Germany). The samples were then incubated for 3 h at 30 °C under static conditions with occasional vortexing. The released cells were harvested. Total RNA was purified using a RNeasy Mini Kit (Qiagen, Hilden, Germany) according to the manufacturer’s protocol. The sequencing libraries were prepared according to the TruSeq RNA protocol (Illumina) by BioNanoPark Łódź, Poland. Sequencing was performed in the pair-end mode (75 cycles) using a NextSeq500 sequencer (Illumina). Between 17 and 20 million reads were generated per library. The raw reads were deposited at GenBank under BioProject ID: PRJNA498189.

### Bioinformatic analysis

#### Genome sequence and its annotation

The *K. xylinus* E25 genome (sequence version NZ_CP004360.1) was retrieved with annotation from the NCBI database (access date, May 2017). The presence of signal peptides was predicted using SignalP (v. 4.1; (Petersen et al. 2011)). Transmembrane proteins were predicted using the Phobius web server (access date, June 2017 (Käll et al. 2004, 2007)). The metabolic pathways were drawn and annotated in PathVisio (v. 3.3.0; (van Iersel et al. 2008; Kutmon et al. 2015)).

#### Read mapping and differential gene expression analysis

The sequencing reads were mapped to the *K. xylinus* E25 genome using Bowtie2 (v. 2.3.2; (Langmead and Salzberg 2012)) and the SAM files were compressed to BAM files using SAMtools (v. 1.5; (Li et al. 2009)). The mapped reads were further processed in R (v. 3.4.1) using Rsamtools (v. 1.28.0; (Morgan et al. 2017)), GenomicAlignments (v. 1.12.1, (Lawrence et al. 2013)), GenomicFeatures (v. 1.28.4, (Lawrence et al. 2013)) and Bioconductor packages (Gentleman et al. 2004; Huber et al. 2015). Only reads that mapped to annotated CDS were submitted to further analyses. Differential gene expression analysis was conducted using the DESeq2 (v. 1.16.1, (Love et al. 2014)) R package. Log_2_ fold changes in expression were calculated using DESeq2. Fragments per kilobase per million mapped fragments (FPKM) were calculated using DESeq2 and employed for graphical presentation of the data (bar graphs) only. The results of differential gene expression analysis are presented in Table S2 of Online Resource 2.

#### Functional enrichment

The proteome of *K. xylinus* E25 was annotated using the RAST annotation service (Aziz et al. 2008). In this case, 1384 genes (38%) were annotated with at least one RAST category. Multi-category proteins (assigned to more than one RAST category) were counted in each of their categories. Functional enrichment was conducted using Fisher’s exact test (one-tailed) as implemented in R. The false discovery rate (FDR) was controlled using the Benjamini-Hochberg procedure (Benjamini and Hochberg 1995). The results are presented in Table S3 of Online Resource 2.

## Results

### Growth of *K. xylinus* E25

**Fig. 1:**
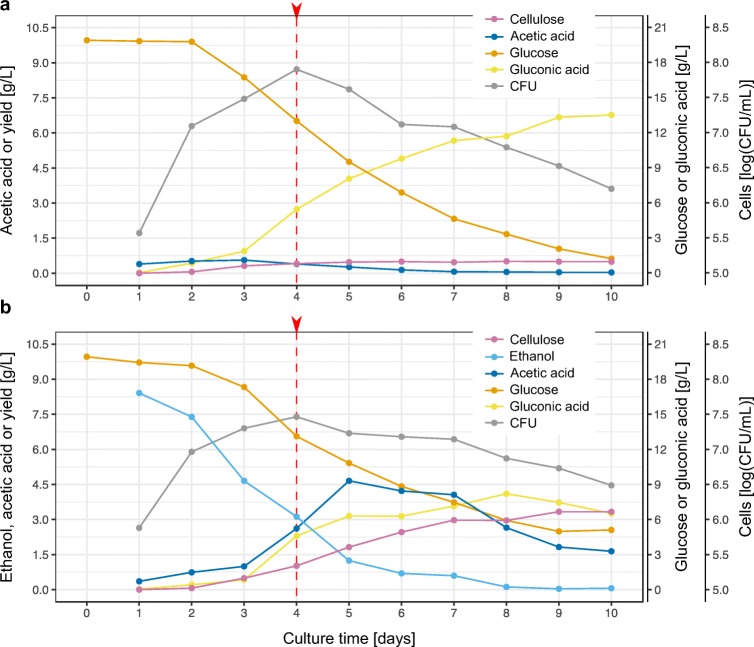
Time course of *K. xylinus* E25 growth in **a** the basal medium and **b** the basal medium supplemented with ethanol. Dots represent mean values from 3 replicated cultures. The red arrow and dashed line mark the fourth day, when cells were harvested for RNA extraction. Graphs displaying pairwise comparisons between the measurements conducted in the two conditions together with standard error values of mean estimates are available in Fig. S1 of Online Resource 1

In the culture broth supplemented with EtOH, *K. xylinus* E25 consumed the ethanol almost completely within 5–6 days (Fig. 1b). In the same culture, acetic acid was accumulated until day 5, after which the concentration decreased slowly. After the first 4 days, the levels of glucose and gluconic acid as well as the cell numbers (expressed as CFU) were similar in both culture broths, whether supplemented with EtOH or not (Fig. 1a, b; *p* value > 0.01 in each case). On the other hand, on day 4, the cellulose yield was significantly greater for the culture containing ethanol (*p* value = 0.0003). After 10 days, the cellulose yield was almost 7 times higher in the medium containing ethanol in comparison to the basal medium (*p* value = 0.0006). The final glucose concentration was significantly higher in the medium containing ethanol (*p* value = 0.0018). The opposite was true for gluconic acid (*p* value = 0.0025). At the end of the culture, the pH of the culture broths was lower, although the drop was significantly smaller in the medium supplemented with ethanol (*p* value = 0.0012; Online Resource 1: Fig. S1a). We also measured the ATP level of cells in both culture broths. The ATP level of the cells in the culture supplemented with ethanol was found to be significantly lower than that of the basal medium starting from day 4 onward (*p* value < 0.005 in each case; Online Resource 1: Fig. S1g).

To explore the molecular changes induced by ethanol that contributed to the increase in cellulose productivity, we profiled the transcriptome of the cells from both culture broths on day 4 using RNA sequencing technology.

### Influence of ethanol on global gene expression in *K. xylinus* E25

Gene expression analysis resulted in the identification of 395 (11.3%) significantly differentially expressed genes (at adjusted *p* value < 0.05 significance threshold). Of these, 129 (3.7%) genes were up-regulated and 266 (7.6%) were repressed. To investigate which functions were overrepresented among the differentially expressed genes (DEGs), we performed enrichment tests in RAST functional groups, for the significantly up-regulated and down-regulated genes separately (Online Resource 2: Table S3 and Online Resource 1: Fig. S2). The general pattern which emerged from this analysis was that ethanol influenced the central metabolism pathways, with a positive effect on protein synthesis and electron accepting reactions, but having a negative impact on glycolysis/gluconeogenesis, TCA cycle, electron donating reactions, protein degradation, and stress response. Minor RAST subsystems, which were significantly enriched included “lactate utilization” (up-regulation), “bacterial hemoglobins,” “rhamnose-containing glycans,” “acetoin,” and “cobalt-zinc-cadmium resistance” (down-regulation).

We next conducted a detailed study of the transcriptional changes in the central metabolism pathways and within the functional groups exposed by the enrichment analysis.

#### Expression of genes involved in ethanol oxidation and TCA cycle progression

**Fig. 2:**
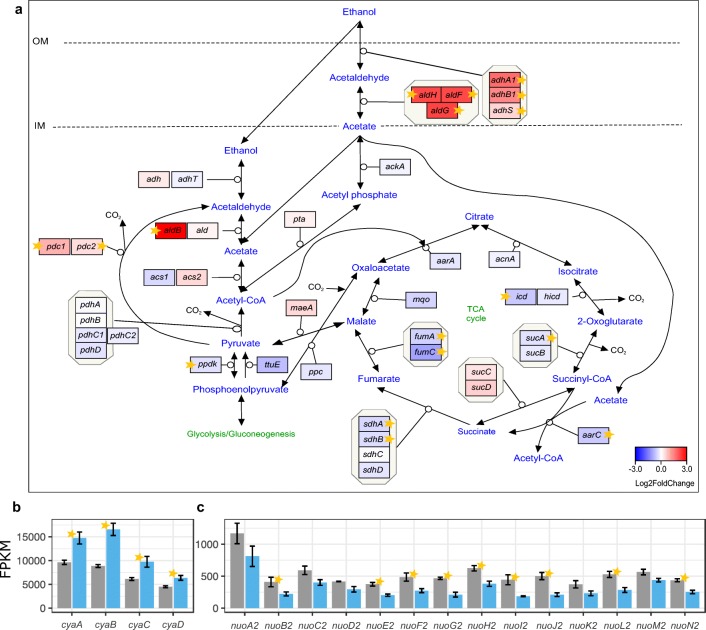
Changes in expression of genes involved in ethanol oxidation and TCA cycle in *K. xylinus* E25. **a** The general pathway of ethanol metabolism and the TCA cycle progression. Genes are colored according to log_2_ fold change in expression between SH and SH+EtOH cultures. Stars denote statistically significant changes (called by DESeq2; adjusted *p* value ≤ 0.05). IM, inner membrane; OM, outer membrane. Mean expression levels (FPKM) of **b** subunits of cytochrome ba(3) ubiquinol oxidase (UOX); **c** subunits of NADH-quinone oxidoreductase, operon 2. Transcripts mean FPKM values are shown in either gray or blue, for cells grown in either the basal medium or the medium supplemented with EtOH, respectively. Bars represent the means from 3 replicated cultures. Thin black bars denote standard error

The measured mRNA levels showed significant induction of genes encoding subunits of membrane-bound alcohol (ADH) and aldehyde dehydrogenase (ALDH) in cells grown on ethanol (Fig. 2a). Moreover, the results suggest that by day 4, ethanol turnover by cytosolic alcohol dehydrogenase had not been induced (Fig. 2a). The acetate accumulated due to the activity of periplasmic enzymes was probably not yet utilized, since the acetate kinase (*ackA*) and the succinyl-CoA:acetate CoA-transferase (*aarC*) genes were down-regulated (Fig. 2a). In the case of the *aarC* gene, there was significant repression (Online Resource 1: Fig. S3b). Unexpectedly, the gene encoding the NADP-dependent ALDH subunit *aldB* as well as both gene copies encoding pyruvate decarboxylase (*pdc*) experienced significant induction (Fig. 2a). Furthermore, the genes involved in TCA cycle progression were mostly repressed, often significantly (Fig. 2a and Online Resource 1: Fig. S3).

Oxidation of ethanol in the periplasm by membrane-bound ADH and ALDH is coupled to the reduction of ubiquinone to ubiquinol. Indeed, we observed significant induction of the expression of genes encoding all subunits of the ba_3_-type ubiquinol oxidase (Fig. 2b). On the other hand, the expression of the *nuo2* operon of NADH dehydrogenase (complex I) was significantly down-regulated in cells grown in the medium with ethanol (Fig. 2c).

#### Expression of the glycolysis/gluconeogenesis pathway

**Fig. 3:**
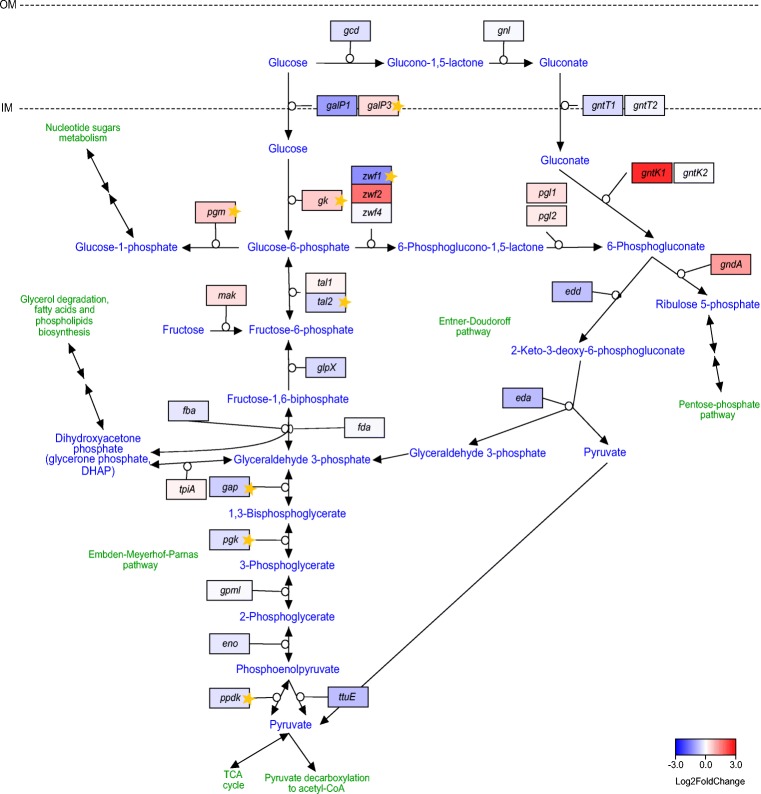
Changes in expression of genes involved in glucose transport and glycolysis/gluconeogenesis. Genes are colored according to log_2_ fold change in expression between SH and SH+EtOH cultures. Stars denote statistically significant changes (called by DESeq2; adjusted *p* value ≤ 0.05). IM, inner membrane; OM, outer membrane

Before entering a cell, glucose may already be oxidized in the periplasm by the action of quinoprotein glucose dehydrogenase (PQQ-GDH). The expression of the gene encoding this enzyme (*gcd*), as well as of the two predicted isoforms of the high-affinity gluconate transporter (*gntT1* and *gntT2*), was mildly down-regulated in the presence of ethanol (Fig. 3). In our previous work, we suggested that the main glucose transporters in the cytoplasmic membrane are the homologs of the *Escherichia coli galP* gene (Ryngajłło et al. 2018). Interestingly, the expression of *galP3*, the most highly expressed isoform, was significantly induced in cells grown in the ethanol-supplemented medium (Fig. 3). Furthermore, the expression of the enzyme responsible for glucose phosphorylation, glucokinase (*gk*), was significantly up-regulated (Fig. 3). This suggests that in cells grown in the medium supplemented with ethanol, the preferred route for glucose is intake and phosphorylation in the cytosol rather than conversion to gluconate in the periplasm. Glucose-6-phosphate may be further degraded in a glycolytic pathway, converted to 6-phosphoglucono-1,5-lactone or to glucose-1-phosphate (G1P). However, conversion to G1P seems most likely since the expression of the second isoform of transaldolase *tal2*, which was the most highly expressed gene copy, was repressed significantly (Fig. 3). Moreover, the two isoforms of glucose-6-phosphate 1-dehydrogenase (*zwf*) were down-regulated (Fig. 3). There was significant repression in the case of *zwf1*. On the other hand, the expression of the phosphoglucomutase gene (*pgm*) was significantly induced (Fig. 3).

The genome of *K. xylinus* E25 harbors genes involved in the two variant pathways of glycolysis, the Embden-Meyerhof-Parnas (EMP) and the Entner-Doudoroff (ED) (Fig. 3). The majority of genes from these pathways were down-regulated. Moreover, for genes such as glyceraldehyde-3-phosphate dehydrogenase (*gap*), phosphoglycerate kinase (*pgk*) and pyruvate, and phosphate dikinase (*ppdk*) from the EMP pathway, this negative change was significant (Fig. 3). The mild up-regulation of both 6-phosphogluconolactonase gene copies (*pgl1* and *pgl2*), thermoresistant gluconokinase (*gntK1*) and NADP(+)-dependent 6-phosphogluconate dehydrogenase (*gndA*), suggest a slight increase in the activity of the first part of the PPP in the presence of ethanol (Fig. 3). However, the second part of the PPP, which leads to the formation of glyceraldehyde 3-phosphate, was generally repressed (Online Resource 1: Fig. S4).

These results suggest that the glucose metabolism of cells grown in the medium supplemented with ethanol was induced in the direction of nucleotide sugars synthesis, rather than along the catabolic pathways of glycolysis or the pentose phosphate pathway.

#### Expression of EPS biosynthesis and c-di-GMP metabolism pathways

**Fig. 4:**
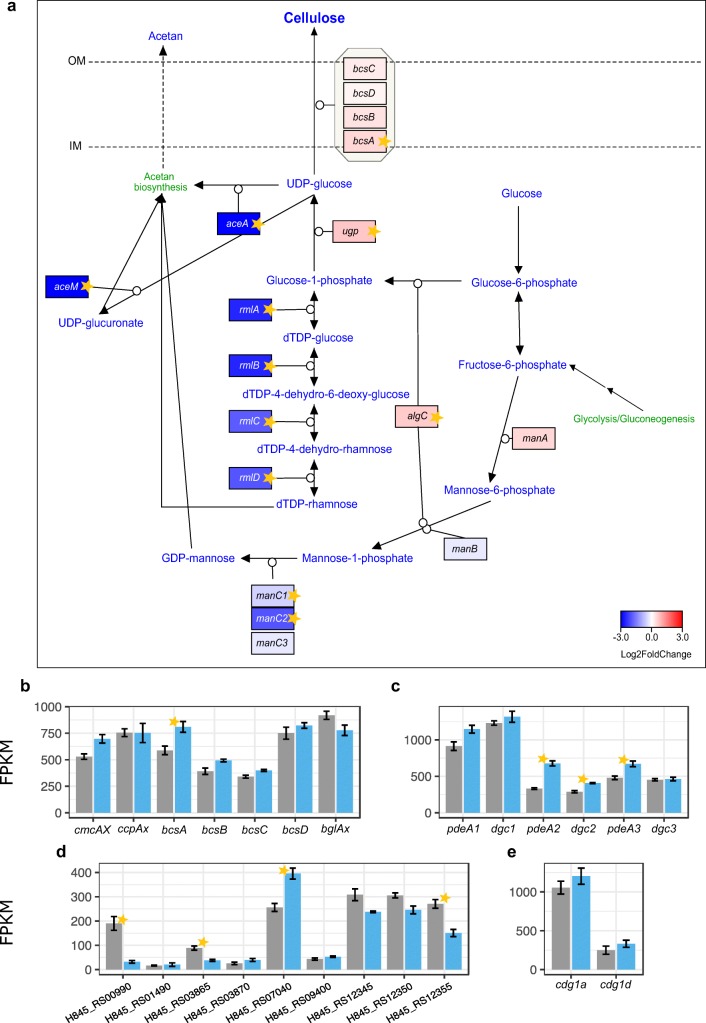
Expression of EPS biosynthesis and c-di-GMP metabolism pathways. **a** Pathway of cellulose and acetan biosynthesis. Genes are colored according to log_2_ fold change in expression between SH and SH+EtOH cultures. IM, inner membrane; OM, outer membrane. Stars denote statistically significant changes (called by DESeq2; adjusted *p* value ≤ 0.05). **b** Expression of genes encoding cellulose biosynthesis operon. **c** Expression of genes encoding the three canonical operons of c-di-GMP turnover enzymes. **d** Expression of genes encoding other GGDEF-EAL proteins. **e** Expression of the accessory genes of the *cdg1* operon, which encode putative transcriptional regulators. Transcripts mean FPKM values are shown in either gray or blue, for cells grown in either the basal medium or the medium supplemented with EtOH, respectively. Bars represent the means from 3 replicated cultures. Thin black bars denote standard error

When ethanol was present in the SH medium, the expression of UDP-glucose pyrophosphorylase was significantly up-regulated (Fig. 4a), which suggests enhanced UDP-glucose synthesis. Furthermore, all genes in the *bcs* operon were up-regulated and there was a significant induction of *bcsA* (Fig. 4a, b). Of the genes flanking the *bcs* operon, only the gene encoding endo-β-1,4-glucanase (*cmcAX*) was up-regulated (Fig. 4b).

In contrast to the pathway of cellulose production, the acetan biosynthesis pathway was down-regulated in cells grown in the medium with ethanol (Fig. 4a). The *aceA* gene, which is the priming enzyme for acetan biosynthesis, as well as the majority of genes involved in the synthesis of its nucleotide sugar precursors (UDP-glucuronate, GDP-mannose, dTDP-rhamnose) were significantly down-regulated (Fig. 4a and Online Resource 1: Fig. S5). The down-regulation of these pathways should increase the UDP-glucose pool and may direct it mainly towards cellulose biosynthesis. Moreover, it is likely that the significant up-regulation of the *algC* gene, which encodes bifunctional phosphomannomutase/phosphoglucomutase, could lead to increased glucose-1-phosphate synthesis since the GDP-mannose pathway was generally repressed. These results imply that when ethanol is present in SH medium, the pathway towards cellulose synthesis is induced, whereas the acetan biosynthesis pathway is strongly down-regulated.

In our previous work, we predicted the presence of 15 genes putatively related to c-di-GMP turnover in the *K. xylinus* E25 genome (Ryngajłło et al. 2018). The three canonical *cdg* operons were indeed found to be the most highly expressed of those genes (Fig. 4c, d), which is consistent with the results of proteomic studies (Tal et al. 1998). In cells grown in the medium supplemented with ethanol, expression of the *cdg* operons was up-regulated, with significant changes in the cases of *cdg2* and *cdg3* (Fig. 4c). Expression of another two genes encoding putative GGDEF-EAL proteins, H845_RS00990 and H845_RS07040, was significantly differentially regulated (Fig. 4d). On the other hand, two genes which were significantly down-regulated may encode transmembrane GGDEF-EAL proteins (H845_RS03865 and H845_RS12355; prediction made using Phobius; Fig. 4d). We observed mild induction of the *cdg1a* and *cdg1d* genes of the first operon, which encodes putative transcriptional regulators (Fig. 4e). The mRNA levels of *cdg1a*, which encodes the Crp-Fnr transcriptional factor, was at the same level as those of *dgc1* and *pde1*, whereas *cdg1d*, which encodes a repressor of the Rrf2 family, was expressed at a much lower level. The substantially lower level of *cdg1d* mRNA in comparison to other genes of the *cdg1* operon suggests that it might be post-transcriptionally down-regulated.

Taken together, these results show that ethanol induced c-di-GMP metabolism at the transcriptional level.

#### Expression of genes involved in lactate and acetoin metabolism

**Fig. 5:**
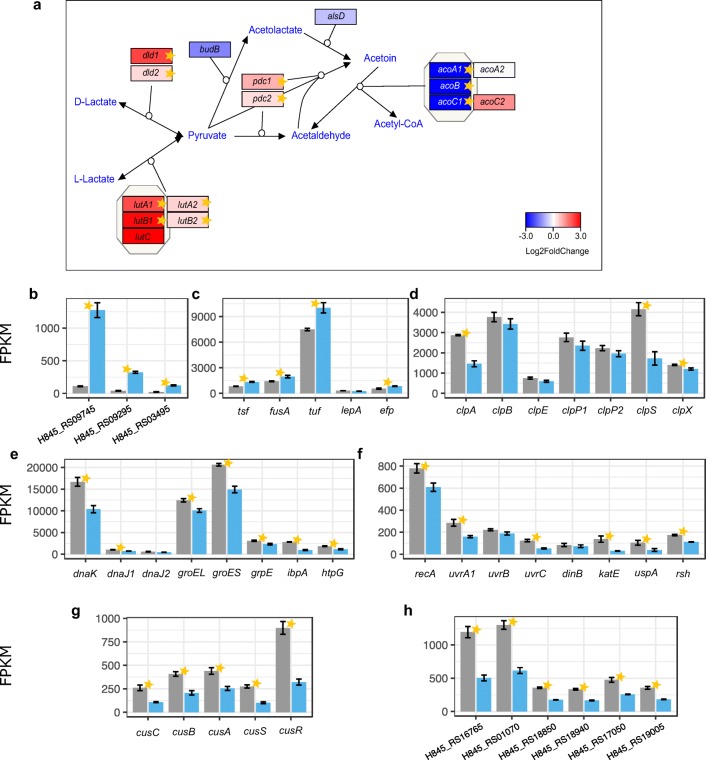
Changes in the expression of genes from various functional groups. **a** The putative pathway of lactate and acetoin metabolism in *K. xylinus* E25. Genes are colored according to log_2_ fold change in expression between SH and SH+EtOH cultures. Stars denote statistically significant changes (called by DESeq2; adjusted *p* value ≤ 0.05). **b** Expression of genes encoding the predicted TonB-dependent receptors (shown are only the genes, which undergo significant changes in expression). **c** Expression of genes encoding translation elongation factors. **d** Expression of genes encoding ATP-dependent Clp proteases. **e** Expression of various molecular chaperons. **f** Expression of various stress-related genes. **g** Expression of genes encoding the predicted copper or cobalt, zinc, and cadmium efflux system. **h** Expression of genes encoding IS*110* family transposases (shown are only the genes, which undergo significant changes in expression). Transcripts mean FPKM values are shown in either gray or blue, for cells grown in the basal medium or the medium supplemented with EtOH, respectively. Bars represent the means from 3 replicated cultures. Thin black bars denote standard error

The results of functional enrichment suggest that, in cells grown on ethanol, lactate utilization may have been activated (Online Resource 1: Fig. S2). *K. xylinus* E25 has two sets of genes, which encode putative D- or L-lactate dehydrogenases. The *dld*, *lutA*, and *lutB* genes and their paralogs were indeed significantly induced in the cells grown in the ethanol-supplemented medium (Fig. 5a and Online Resource 1: Fig. S6). The activation of lactate dehydrogenase genes was unexpected since lactate was absent in SH medium. It is possible that ethanol has a regulatory effect on the lactate turnover pathway.

Furthermore, functional enrichment analysis of the RAST subsystems highlighted acetoin metabolism as a particularly repressed pathway. The genome of *K. xylinus* E25 carries the predicted acetoin metabolism genes, which suggests that the bacterium synthesizes that metabolite. However, the genes encoding acetolactate synthase (*budB*) and alpha-acetolactate decarboxylase (*alsD*) were negatively regulated in the ethanol-containing medium (Fig. 5a). Acetoin may also be generated by pyruvate decarboxylase as a by-product (Wu et al. 2000). As discussed above, the *pdc* genes were significantly up-regulated, which may suggest that their products play a role in the process of acetoin accumulation in cells grown in the presence of ethanol. Moreover, the genes encoding the putative acetoin dehydrogenase complex, comprising the first *acoABC* operon, were very strongly and significantly repressed (Fig. 5a; Online Resource 1: Fig. S7). Taken together, these results suggest that the presence of ethanol in the medium activates acetoin synthesis, while simultaneously inhibiting its catabolism.

#### Iron uptake

Among the genes that displayed particularly contrasting levels of expression in the basal and the ethanol-supplemented media, we found TonB-dependent receptors. The majority of the 28 genes present in the genome of *K. xylinus* E25 were up-regulated (Online Resource 1: Fig. S8), with 3 induced significantly (Fig. 5b). Although the function of the most highly expressed gene of this group, H845_RS09745, is unknown, the majority of the genes share sequence similarity with siderophore receptors (ferrichrome, Fe^3+^ citrate, ferric enterobactin). The H845_RS03495 gene is a homolog of the hemin receptor *hmuR* of *Yersinia pestis*. Interestingly, this gene is arranged in a cluster with four other genes (H845_RS03500–H845_RS03510) and they all share sequence similarity with the *hmuRTUV* cluster of *Y. pestis*, which is a known hemin uptake locus characterized in this bacterium (Thompson et al. 1999). The expression of the genes in this cluster was up-regulated when ethanol was present in the culture medium (Online Resource 1: Fig. S9a). The presence of the hemin uptake locus suggests that *K. xylinus* E25 has the ability to scavenge iron from heme or heme-containing proteins. We observed no induction in the expression of genes involved in de novo heme biosynthesis (Online Resource 1: Fig. S9b). Moreover, a gene predicted to encode porphobilinogen synthase (H845_RS08335) was significantly down-regulated. These results imply that when ethanol is present in the medium, cells prefer to import heme from the medium rather than perform heme biosynthesis.

#### Protein metabolism, molecular chaperons, stress-induced genes, and metal efflux system

The results for functional enrichment appeared to indicate particular induction of pathways connected to protein biosynthesis (Online Resource 1: Fig. S2). Indeed, we observed that the majority of genes encoding subunits of ribosomal proteins were induced in cells grown in the ethanol-supplemented medium (Online Resource 1: Fig. S10). Moreover, most of the genes encoding translation elongation factors were significantly up-regulated (Fig. 5c). In contrast, proteins involved in protein degradation, such as ATP-dependent proteases, were repressed, often significantly (Fig. 5d and Online Resource 1: Fig. S11). These results demonstrate that the addition of ethanol to the growth medium had a positive influence on protein biosynthesis while repressing protein degradation.

Since “stress response” was one of the significantly enriched RAST categories among the repressed genes (Online Resource 1: Fig. S2), we decided to investigate the expression of genes related to this group. Our results imply that the majority of molecular chaperons and stress-related genes were significantly down-regulated in cells grown on the ethanol-supplemented medium in comparison to those grown on the basal medium (Fig. 5e, f). Moreover, the results of functional enrichment analysis revealed the genes involved in cobalt, zinc, and cadmium metal resistance as a particularly repressed subsystem. Upon closer inspection, this group was seen to include a cluster of genes which share sequence similarity with known metal ion efflux systems, such as the *czc* operon of *Ralstonia eutropha* (formerly *Alcaligenes eutrophus*) strain CH34, which provides resistance to cobalt, zinc, and cadmium, or the *cus* operon of *E. coli* K-12, which encodes the detoxification system for copper ions (Nies et al. 1989; Munson et al. 2000). In the medium supplemented with ethanol, the *cusCBASR* genes in *K. xylinus* E25 were significantly down-regulated (Fig. 5g). The induction of these genes in cells grown in SH medium suggests that toxic levels of metals accumulated, probably causing cellular stress and activating their efflux systems. Moreover, the list of significantly repressed genes was found to be particularly enriched in those genes that encode transposases of the IS*110* family (Fig. 5h and Online Resource 1: Fig. S12). These transposases, enclosed within insertion sequences, are located either on the pGX4 or the pGX5 plasmid. This result is particularly important since the activation of transposases has usually been associated with cellular stress. The exact role of IS*110* in *Komagataeibacter* remains, however, unclear. Collectively, our analysis of gene expression shows that the bacteria grown in SH medium deprived of ethanol experienced higher cellular stress than those in the ethanol-supplemented medium.

## Discussion

It has long been recognized that the addition of ethanol significantly increases BNC yields from several strains of the *Komagataeibacter* genus (Naritomi et al. 1998b; Krystynowicz et al. 2002; Park et al. 2003; Dubey et al. 2017; Liu et al. 2018; Molina-Ramírez et al. 2018). Given the spectacular influence of ethanol on cellulose synthesis, efforts have been made to better understand its molecular basis. An insight into the process has been provided by studies investigating changes at the metabolite and protein levels (Naritomi et al. 1998b; Kornmann et al. 2003), as well as in research employing ^13^C-labeled carbon (Arashida et al. 1993; Yunoki et al. 2004). However, to the best knowledge of the authors, no previous global study has been made of the systemic cellular response to the presence of ethanol in the culture medium. The modulation of gene expression is one of the most rapid ways by which cells adjust to new environmental conditions. Therefore, to improve understanding of the global effect of ethanol addition on cellular functions, we sequenced and compared the levels of total RNA in cells grown on medium supplemented with ethanol and the control, SH medium.

Our results show that, on the fourth day of growth, the cells actively utilized ethanol and accumulated acetate. The transcriptomic results agreed well with the metabolite profiles since significant induction of periplasmic alcohol and aldehyde dehydrogenases was observed. Moreover, our results suggest that the acetate accumulating state was caused by the inhibition of genes encoding acetate kinase, succinyl-CoA:acetate CoA-transferase, and TCA cycle enzymes. This is in accordance with previous observations, which had shown that the presence of ethanol attenuates carbon flux through the TCA cycle in *K. xylinus* I-2281 (Kornmann et al. 2003) and in *Acetobacter* strains (Sakurai et al. 2011; Adler et al. 2014). Accordingly, the majority of genes encoding the NADH dehydrogenase complex were significantly repressed. Based on similar observations in the case of *Acetobacter aceti* NBRC 14818, it has been suggested that the low induction of NADH dehydrogenase genes in ethanol-supplemented medium is probably the result of direct ubiquinone reduction by periplasmic ADH and ALDH enzymes (Sakurai et al. 2011). The enzymes, which oxidize acetate in the cytosol and generate NADH, probably become activated during the later growth phase. One exception was the induction of the NADP-dependent ALDH subunit *aldB*. This gene has previously been reported to be induced by ethanol in *E. coli* (Kwok and Weiner 2005). Its role may be to remove alcohols and aldehydes from stressed cells. It is possible that the *aldB* homolog functions similarly in *K. xylinus* E25 and may already be induced in the late ethanol oxidation phase.

The induced membrane-bound alcohol and aldehyde dehydrogenases should reduce ubiquinone more actively. Therefore, it was not surprising to observe up-regulation of the genes encoding ubiquinol oxidase. Importantly, the up-regulation of ubiquinol oxidase genes suggests an increase in oxygen reduction, which should have a direct and positive effect on cellular energetics. However, the measured level for ATP was much lower in the cells grown in the ethanol-supplemented medium. One possible explanation for the lower intracellular ATP content could be increased ATP consumption during growth on the ethanol medium. Several acetic acid resistance mechanisms have been reported in AAB (Nakano and Ebisuya 2016). One of the putative ways by which AAB cells prevent its intracellular accumulation is by exporting acetic acid via an ABC transporter and/or a proton motive force-dependent efflux pump. It is possible that the energy generated during ethanol oxidation, either in the form of ATP or a proton motive force, is used to remove acetic acid from the cytosol. Moreover, increased consumption of energy may result from induced import of medium nutrients. What is more, the export of large amounts of cellulose is likely to drain on cellular energy severely. The low level of ATP produced by cells grown in the ethanol-supplemented medium may be also due to down-regulation of the TCA cycle. Our observations stand in contrast to a study of *Komagataeibacter sucrofermentans* BPR3001A, in which higher ATP levels were reported for a culture grown in a medium supplemented with ethanol (Naritomi et al. 1998b). This discrepancy may be due to the difference in the culturing conditions since a continuous culture in a bioreactor with agitation was employed in that study.

Another conclusion which may be drawn from our results is that ethanol attenuates glucose consumption and gluconate production. Studies of *K. xylinus* ATCC 10245 and *A. aceti* revealed a similar decrease in the glucose consumption rate in a medium supplemented with ethanol (Yunoki et al. 2004; Sakurai et al. 2011). The transcript profiles gathered in our study provide a plausible explanation for the molecular changes which negatively influence glucose consumption and gluconate production. First of all, we observed that when *K. xylinus* E25 was actively oxidizing ethanol, the genes related to gluconate synthesis and transport and to glycolysis were generally repressed. It has been suggested that increased levels of ATP in cells growing on ethanol inhibit glucose-6-phosphate dehydrogenase enzyme in *K. sucrofermentans* BPR3001A (Naritomi et al. 1998b). Our results imply that negative regulation of this enzyme already occurs at the transcriptional level. Despite the observed general inhibition of the glucose degradation pathways, our results suggest that the route leading to cellulose formation, which involves the putative glucose transporter (*galP3*), glucokinase, UDP-glucose pyrophosphorylase, phosphoglucomutase, and the *bcsABCD* operon, was induced.

It has been postulated that the increased cellulose yield associated with the presence of ethanol in the medium may be linked to a more effective use of glucose for cellulose synthesis since the glucose is not degraded for energy acquisition (Yunoki et al. 2004). Our gene expression-based findings support this conclusion. What is more, the pathway towards acetan formation, which competes with cellulose synthesis for nucleotide sugar precursors, was strongly repressed. These expression results correspond to what had previously been observed in a study of *K. xylinus* I-2281 (*Gluconacetobacter* in the original work), in which gluconacetan synthesis was inhibited when ethanol was present in the culture medium (Kornmann et al. 2003). It is possible that ethanol has a similarly inhibitory effect on acetan biosynthesis in *K. xylinus* E25. Interestingly, a negative relationship has been reported between acetan and cellulose biosynthesis (Valla and Kjosbakken 1982; Watanabe et al. 1998). Wild-type cells produce both cellulose and acetan; in certain natural mutant strains, cellulose synthesis is activated concurrently with the inhibition of acetan production. However, the direct deactivation of the priming enzyme for acetan biosynthesis (*aceA*) resulted in a decreased BNC yield, which was likely due to the reduced viscosity of the growth medium (Ishida et al. 2002). It is therefore possible that a minimum level of acetan is necessary and its production cannot be entirely prevented for efficient cellulose synthesis. Our results show that, although the pathway towards acetan biosynthesis was down-regulated, the associated genes were still expressed, which suggests that this EPS was still produced. However, the regulatory mechanism which is responsible for the attenuation of acetan biosynthesis during growth on the ethanol-supplemented medium has yet to be elucidated.

Although our results show that the genes encoding cellulose synthase subunits were only mildly up-regulated in the cultures supplemented with ethanol, we observed significant changes in the mRNA levels of genes encoding the turnover enzymes of c-di-GMP, the main activator of BcsA. The expression level of the main operon (*cdg1*) was unchanged in the control and the ethanol-supplemented medium. However, the mRNA level of the other two operons increased. Interestingly, studies on *E. coli* had shown that many of the c-di-GMP turnover genes are differentially regulated by the global regulator RpoS, in response to growth phase and changes in environmental stimuli (Sommerfeldt et al. 2009). It is likely that ethanol indirectly influences the expression of genes encoding GGDEF-EAL proteins through a global transcriptional regulator such as RpoS. However, given that the functions of the majority of GGDEF-EAL proteins in *K. xylinus* E25 are unknown, it is impossible to speculate on the influence of these transcriptional changes on cellulose biosynthesis and further research is needed to explain their role in the process.

We observed high induction of genes encoding lactate dehydrogenases and strong repression of the acetoin degradation pathway. Studies on three *Acetobacter* species grown on a culture medium containing both ethanol and lactate concluded that acetate is exclusively derived from ethanol, whereas lactate mainly contributes to acetoin and biomass (Adler et al. 2014). Adler et al. observed that lactate was metabolized until ethanol was present in the medium. This may suggest that ethanol activates lactate metabolism. It is possible that in *K. xylinus* E25, lactate turnover is part of the ethanol-dependent regulatory pathway. Indeed, several species of the *Komagataeibacter* genus have been reported to grow in a microbial consortium where lactic acid bacteria and yeast were present (Entani and Masai 1985). Various microorganisms secrete acetoin when they are grown on medium containing glucose, to temporarily store carbon and prevent over-acidification (Xiao and Xu 2007). The metabolite is later degraded in the stationary phase when glucose concentration is low. Our results suggest that acetoin degradation was active in the culture grown in the basal medium and inhibited in the cells grown in the medium supplemented with ethanol. It is possible that less acetoin accumulated in the culture grown on the ethanol medium, since glycolysis was repressed in these cells, and its catabolism was probably therefore not activated. Another possibility is that acetoin metabolism is connected to the ethanol-dependent regulatory pathway, which activates lactate consumption. The conversion of lactate to acetoin, instead of pyruvic acid, may prevent over-acidification when acetic acid accumulates. More research is needed, however, to better understand these regulatory circuits.

Further analysis revealed that the cells grown in the ethanol-supplemented medium were in a state that promoted cellular growth, since the genes encoding ribosomal proteins and translation elongation factors were mostly significantly induced, in contrast to those related to protein degradation. Activation of protein biosynthesis may be due to the preparation of the cells for the second growth phase, connected to acetic acid oxidation in the cytosol. This possibility seems likely since such a metabolic switch would require the synthesis of a different set of enzymes. One of the micronutrients essential for bacterial growth is iron since it is required for key biological processes such as amino acid synthesis, the TCA cycle, oxygen transport, and respiration. Interestingly, we observed the induction of the expression of genes encoding TonB-dependent receptors, which may be involved in iron intake. Our findings also show that when ethanol was present in the medium, instead of synthesizing it, the cells actively imported heme from the medium. Based on these findings, it may be concluded that the energy produced during ethanol oxidation was utilized for the active transport of nutrients from the culture media, which probably had a positive effect on the maintenance of cellular growth and BNC biosynthesis. This view is supported by an analysis employing isotope-labeled carbon in *K. xylinus* ATCC 10245, which showed that ethanol-induced gluconeogenesis from carbon sources, such as amino acids or polypeptides contained in peptone and yeast extract, in the culture medium (Yunoki et al. 2004).

In a study of *A. aceti*, it was observed that molecular chaperons and genes for the SOS response system were induced in an ethanol-supplemented medium (Sakurai et al. 2012). However, our results for gene expression show that the *K. xylinus* E25 cells were not particularly stressed during ethanol oxidation since various stress-related genes were mostly significantly repressed. It is possible that the efflux system managed to efficiently pump the acetic acid out from the cytosol and thus prevented cellular damage. The further metabolism of acetate in the cytosol probably does provoke a stress response, since increased levels of stress-related proteins have been measured in various *Komagataeibacter* strains during acetic acid fermentation (Andrés-Barrao et al. 2016). What is more, extensive degradation of glucose by the cells grown in the basal medium might result in oxidative stress. Induction of the catalase gene (*katE*) supports the possibility that removal of peroxides was activated. Another factor which could cause cellular stress was the increased level of metals, which is suggested by the up-regulated cobalt, zinc, and cadmium detoxification system in the cells grown in the SH medium.

Finally, we observed significant repression of IS*110* transposases in the ethanol-supplemented medium, in comparison to the basal medium. It has been suggested that the presence of ethanol inhibits the formation of cellulose-nonproducing cells (Cel-) in agitated cultures (Son et al. 2001; Krystynowicz et al. 2002, 2005; Park et al. 2003). Interestingly, at the molecular level, the appearance of Cel- cells has been found to often involve the transposition of an indigenous insertion sequence into genes associated with cellulose synthesis (Coucheron 1991; Standal et al. 1994; Matsutani et al. 2015). Thus, the inhibition of transposase gene expression that we observed provides the first plausible explanation for the positive effect of ethanol supplementation on the genotypic stability of a BNC producer, and possibly for the inhibition of Cel- formation. Further studies involving genome sequencing are needed, however, to verify the effect of ethanol-induced attenuation of IS*110* transposition on the phenotype of *K. xylinus* E25.

To our knowledge, this study is the first attempt to evaluate the influence of ethanol on the cellular functions of a *Komagataeibacter* sp. based on transcriptome profiling. The results presented in this work should direct future biochemical and molecular studies, which could improve understanding of genome-phenotype linkage in BNC producers.

## Electronic supplementary material

ESM 1 (PDF 1344 kb)

ESM 2 (XLSX 1105 kb)
