# Supplementary material for: Effect of ethanol supplementation on the transcriptional landscape of bionanocellulose producer Komagataeibacter xylinus E25
Source: Appl Microbiol Biotechnol. 2019 Jun 6;103(16):6673–88. doi: 10.1007/s00253-019-09904-x (PMC6667682; doi:10.1007/s00253-019-09904-x)
Supplement: Supplementary file 1 — (PDF 1344 kb) [file 253_2019_9904_MOESM1_ESM.pdf]

Applied Microbiology and Biotechnology

Effect of ethanol supplementation on the transcriptional landscape of bionanocellulose producer *Komagataeibacter xylinus* E25

Małgorzata Ryngajłło*, Paulina Jacek, Izabela Cielecka, Halina Kalinowska, Stanisław Bielecki

Institute of Technical Biochemistry, Lodz University of Technology, B. Stefanowskiego 4/10, 90-924 Lodz, Poland

*Corresponding author
Email: malgorzata.ryngajllo@p.lodz.pl
Telephone: +48 0426313354
ORCID: 0000-0002-0430-0102

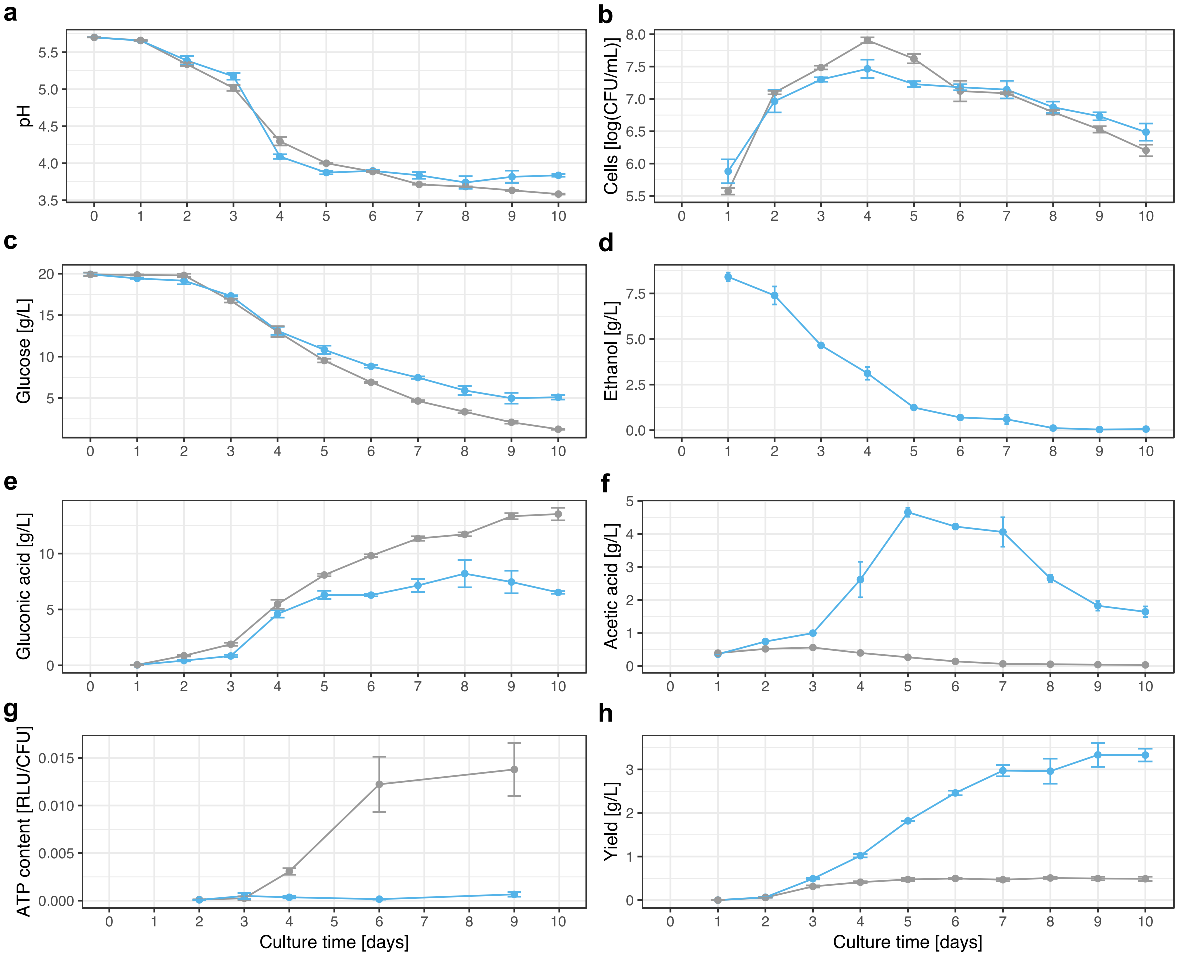

Fig. S1 Changes in pH, cell number, yield, ATP content and level of metabolites of *K. xylinus* E25 growing in the medium supplemented with ethanol (light blue lines) or the basal medium (grey lines). CFU – colony forming units; RLU – relative light units. Dots represent the mean value of 3 replicated cultures. Thin bars denote standard error.

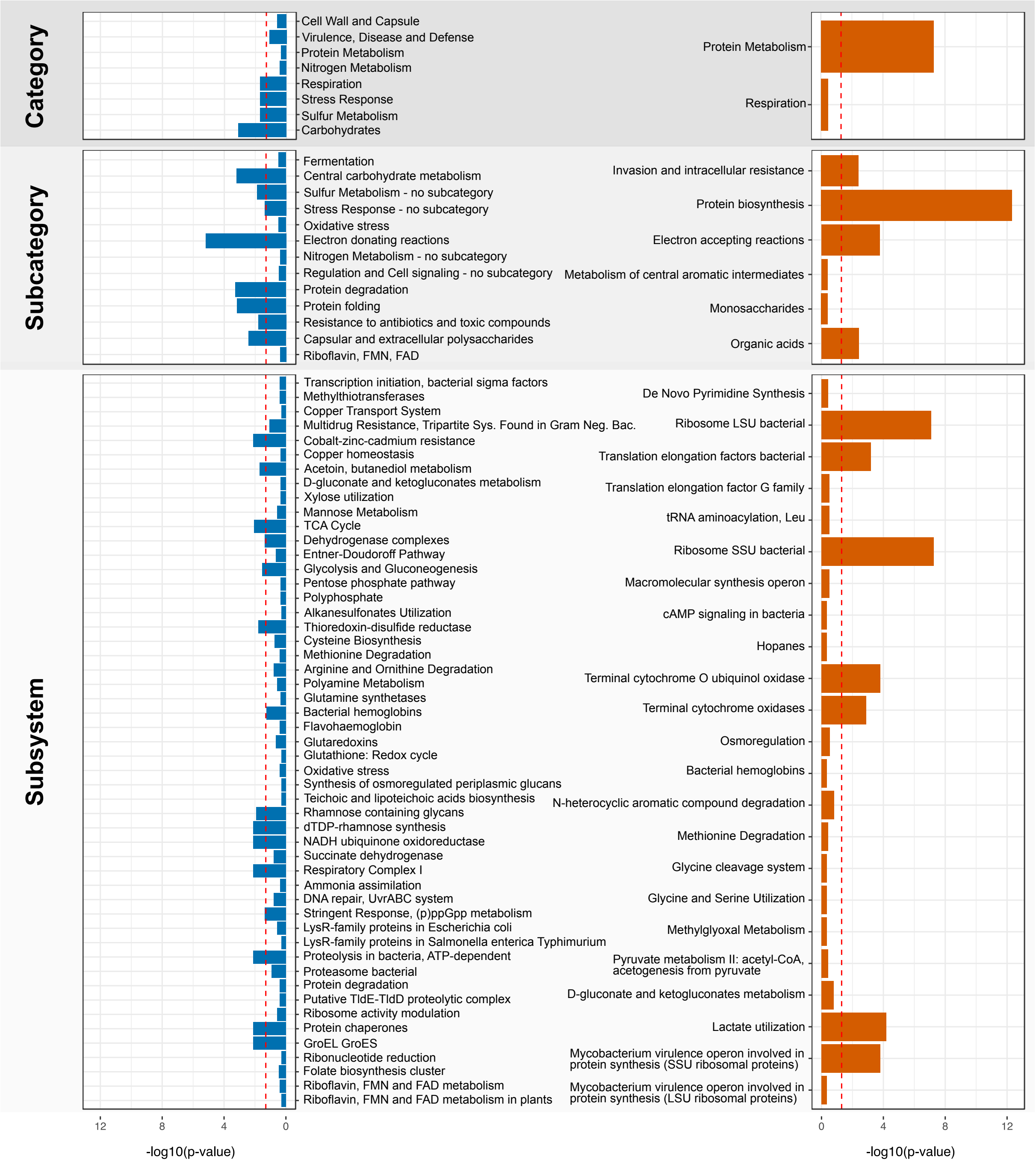

Fig. S2 Functional enrichment analysis. Enriched RAST categories (top panels), subcategories (middle panels), and subsystems (bottom panels) among the down-regulated (blue bars) and up-regulated (orange bars) genes. Shown are results of one-sided Fisher's Exact test (one-tailed). Presented are only bins, which scored the adjusted *p-value* below 0.5. The red, dashed vertical line indicates adjusted *p-value* threshold of 0.05.

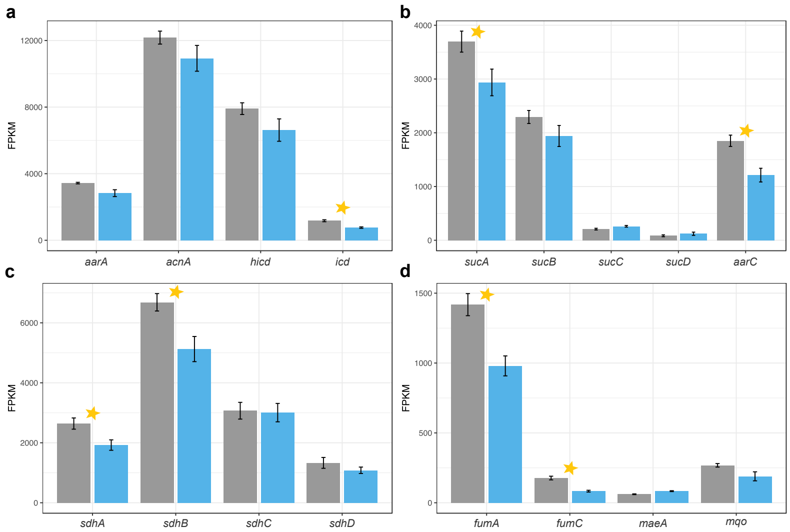

Fig. S3 The mean FPKM values for genes encoding enzymes of the TCA pathway in SH medium (grey) and in the medium supplemented with EtOH (blue). Bars represent the means from 3 replicated cultures. Thin black bars denote the standard error. Yellow stars denote significant changes in expression between the conditions (called by DESeq2; adjusted *p-value* ≤ 0.05).

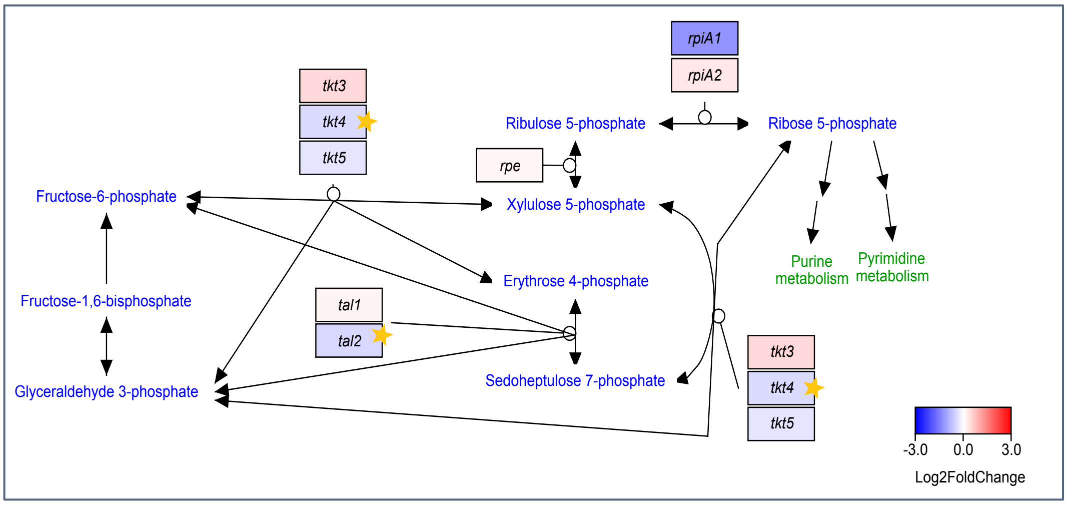

Fig. S4 Changes in expression of genes involved in the pentose phosphate pathway. Genes are coloured according to $\log_2$ fold change in expression between SH and SH+EtOH cultures. Stars denote statistically significant changes (called by DESeq2; adjusted *p-value* ≤ 0.05).

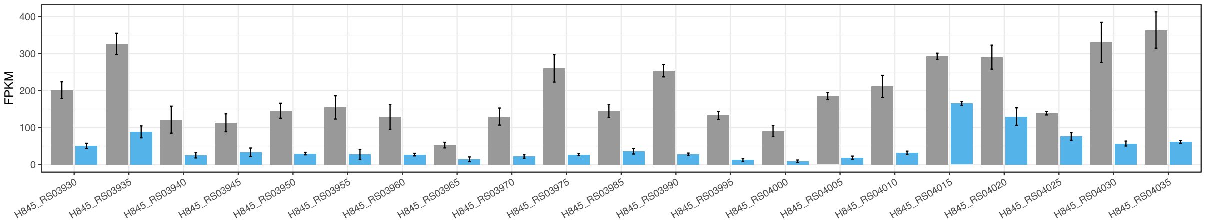

Fig. S5 The mean FPKM values for genes of the acetan biosynthesis cluster the SH medium (grey) and in the medium supplemented with EtOH (blue). Bars represent the means from 3 replicated cultures. Thin black bars denote the standard error. Yellow stars denote significant changes in expression between the conditions (called by DESeq2).

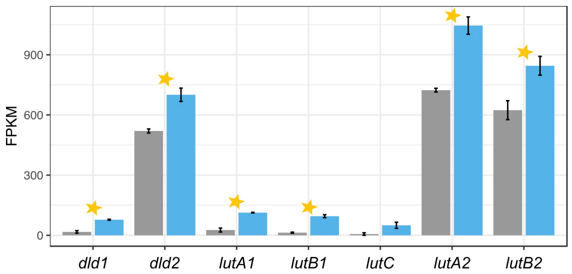

Fig. S6 The mean FPKM values for genes encoding D- and L-lactate dehydrogenases in SH medium (grey) and in the medium supplemented with EtOH (blue). Bars represent the means from 3 replicated cultures. Thin black bars denote the standard error. Yellow stars denote significant changes in expression between the conditions (called by DESeq2; adjusted *p-value* ≤ 0.05).

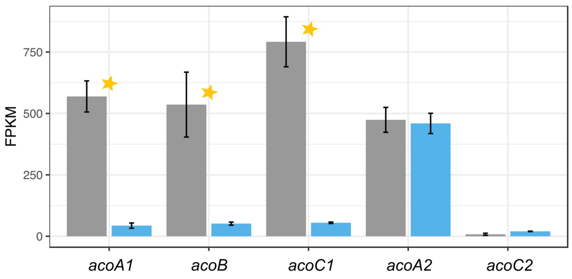

Fig. S7 The mean FPKM values for genes encoding subunits of acetoin dehydrogenase in SH medium (grey) and in the medium supplemented with EtOH (blue). Bars represent the means from 3 replicated cultures. Thin black bars denote the standard error. Yellow stars denote significant changes in expression between the conditions (called by DESeq2; adjusted *p-value* ≤ 0.05).

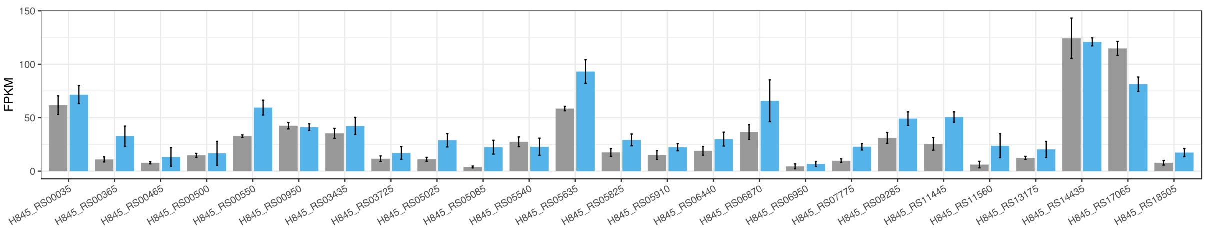

Fig. S8 The mean FPKM values for genes encoding predicted TonB-dependent receptors in SH medium (grey) and in the medium supplemented with EtOH (blue). Bars represent the means from 3 replicated cultures. Thin black bars denote the standard error.

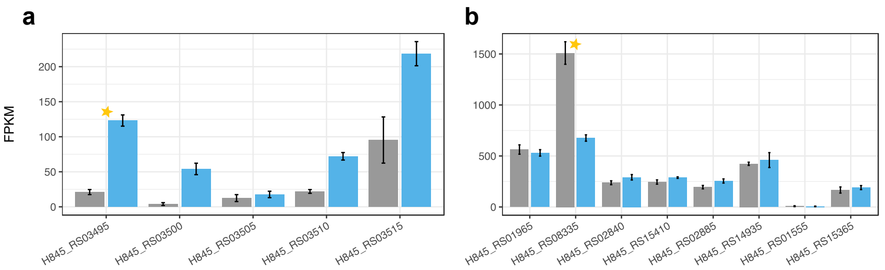

Fig. S9 Mean FPKM values for genes encoding predicted **a).** hemin uptake locus **b).** heme de novo biosynthesis in SH medium (grey) and in the medium with EtOH (blue). Bars represent the means from 3 replicated cultures. Thin black bars denote the standard error. Yellow stars denote significant changes in expression between the conditions (called by DESeq2; adjusted *p-value* ≤ 0.05).

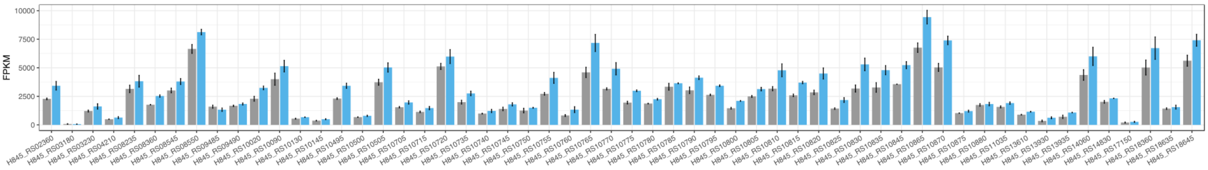

Fig. S10 Expression of genes encoding ribosomal proteins (L and S subunits) in SH medium (grey) and in medium with EtOH (blue). Bars represent average from 3 replicated cultures. Thin black bars denote standard error.

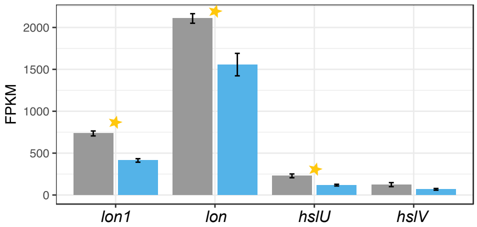

Fig. S11 Expression of various ATP-dependant proteases in SH medium (grey) and in the medium with EtOH (blue). Bars represent the means from 3 replicated cultures. Thin black bars denote standard error. Yellow stars denote significant changes in expression between the conditions (called by DESeq2; adjusted *p-value* ≤ 0.05).

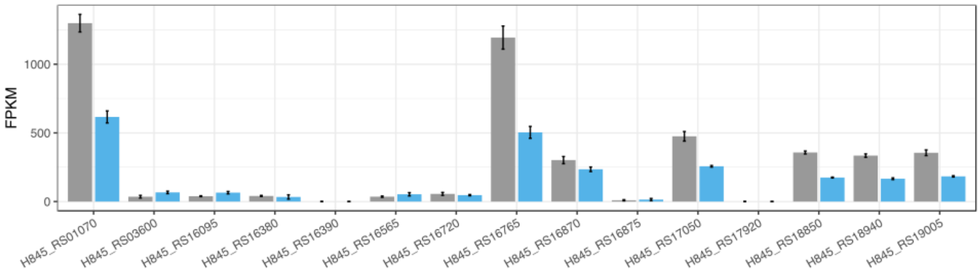

Fig. S12 Mean FPKM values for genes encoding IS110 family transposases in SH medium (grey) and the in medium with EtOH (blue). Bars represent the means from 3 replicated cultures. Thin black bars denote standard error.

Table S1 Results of various growth-related measurements.

	Time [days]	SH					SH+EtOH				
		rep_1	rep_2	rep_3	Avg. SH	SE SH	rep_1	rep_2	rep_3	Avg. SH + EtOH	SE SH + EtOH
Cellulose Yield [g/L]	0	NA	NA	NA	NA	NA	NA	NA	NA	NA	NA
	1	0.00	0.00	0.00	0.00	0.00	0.00	0.00	0.00	0.00	0.00
	2	0.06	0.06	NA	0.06	0.00	0.07	0.08	0.05	0.07	0.01
	3	0.30	0.28	0.36	0.31	0.02	0.52	0.48	0.47	0.49	0.02
	4	0.44	0.37	0.43	0.41	0.02	1.09	0.96	1.01	1.02	0.04
	5	0.41	0.49	0.52	0.47	0.03	1.82	1.81	1.82	1.82	0.00
	6	0.49	0.48	0.52	0.50	0.01	2.36	2.53	2.49	2.46	0.05
	7	0.54	0.44	0.43	0.47	0.03	2.75	2.96	3.21	2.97	0.13
	8	0.52	0.54	0.47	0.51	0.02	2.77	2.58	3.52	2.96	0.29
	9	0.42	0.56	0.51	0.50	0.04	3.57	2.79	3.64	3.33	0.27
	10	0.53	0.39	0.55	0.49	0.05	3.04	3.51	3.44	3.33	0.15
pH	0	5.70	5.70	5.70	5.70	0.00	5.70	5.70	5.70	5.70	0.00
	1	5.67	5.66	5.65	5.66	0.01	5.66	5.66	5.65	5.66	0.00
	2	5.32	5.38	5.31	5.34	0.02	5.29	5.50	5.37	5.39	0.06
	3	4.94	5.06	5.05	5.02	0.04	5.09	5.24	5.19	5.17	0.04
	4	4.23	4.25	4.41	4.30	0.06	4.07	4.05	4.15	4.09	0.03
	5	4.01	4.00	3.99	4.00	0.01	3.83	3.88	3.91	3.87	0.02
	6	3.88	3.90	3.88	3.89	0.01	3.92	3.87	3.90	3.90	0.01
	7	3.71	3.72	3.71	3.71	0.00	3.76	3.83	3.92	3.84	0.05
	8	3.68	3.69	3.68	3.68	0.00	3.65	3.66	3.91	3.74	0.09
	9	3.62	3.64	3.64	3.63	0.01	3.90	3.90	3.65	3.82	0.08
	10	3.57	3.59	3.59	3.58	0.01	3.86	3.80	3.85	3.84	0.02
Glucose [g/L]	0	19.94	19.54	20.28	19.92	0.21	19.94	19.54	20.28	19.92	0.21
	1	19.72	19.94	19.88	19.85	0.07	19.52	19.41	19.36	19.43	0.05
	2	19.39	19.99	20.01	19.80	0.20	19.69	18.29	19.48	19.16	0.44
	3	16.34	16.79	17.13	16.75	0.23	17.22	17.48	17.28	17.33	0.08
	4	12.09	12.71	14.27	13.02	0.65	13.58	13.62	12.15	13.12	0.48
	5	9.89	9.56	9.12	9.52	0.22	10.19	10.48	11.81	10.83	0.50
	6	6.86	7.05	6.81	6.91	0.07	9.14	8.70	8.65	8.83	0.16
	7	4.68	4.80	4.47	4.65	0.10	7.60	7.18	7.63	7.47	0.15
	8	3.64	3.32	3.07	3.34	0.16	5.49	5.27	7.01	5.92	0.55
	9	1.77	2.33	2.16	2.09	0.17	5.58	5.69	3.68	4.98	0.65
	10	1.13	1.34	1.27	1.25	0.06	5.54	4.58	5.19	5.11	0.28
Cells [log(CFU/mL)]	0	NA	NA	NA	NA	NA	NA	NA	NA	NA	NA
	1	5.56	5.67	5.49	5.57	0.05	5.56	6.19	5.89	5.88	0.18
	2	7.10	7.05	7.15	7.10	0.03	7.20	6.63	7.06	6.96	0.17

	3	7.53	7.43	7.49	7.48	0.03	7.37	7.26	7.27	7.30	0.03
	4	7.98	7.92	7.82	7.91	0.05	7.29	7.35	7.75	7.46	0.14
	5	7.51	7.76	7.59	7.62	0.07	7.25	7.29	7.14	7.23	0.05
	6	7.44	6.99	6.93	7.12	0.16	7.27	7.12	7.15	7.18	0.05
	7	7.10	7.05	7.11	7.09	0.02	6.87	7.29	7.27	7.14	0.14
	8	6.85	6.73	6.81	6.80	0.04	7.03	6.85	6.73	6.87	0.09
	9	6.54	6.61	6.44	6.53	0.05	6.66	6.68	6.85	6.73	0.06
	10	6.38	6.16	6.07	6.20	0.09	6.54	6.68	6.23	6.49	0.13
**Ethanol [g/L]**	0	NA	NA	NA	NA	NA	NA	NA	NA	NA	NA
	1	7.94	7.50	NA	7.72	0.22	8.83	8.37	8.02	8.41	0.24
	2	7.61	7.43	NA	7.52	0.09	8.27	7.33	6.57	7.39	0.49
	3	6.25	6.55	NA	6.40	0.15	4.48	4.62	4.86	4.65	0.11
	4	4.59	5.10	NA	4.85	0.25	3.06	2.54	3.75	3.12	0.35
	5	4.32	4.83	NA	4.57	0.25	1.25	1.32	1.16	1.24	0.05
	6	4.52	4.67	NA	4.60	0.08	0.70	0.75	0.64	0.70	0.03
	7	3.11	3.25	NA	3.18	0.07	0.45	0.25	1.09	0.60	0.25
	8	3.08	3.42	NA	3.25	0.17	0.05	0.04	0.26	0.12	0.07
	9	3.08	2.98	NA	3.03	0.05	0.04	0.05	0.01	0.04	0.01
	10	3.28	3.20	NA	3.24	0.04	0.06	0.07	0.05	0.06	0.01
**Acetic acid [g/L]**	0	NA	NA	NA	NA	NA	NA	NA	NA	NA	NA
	1	0.39	0.39	0.39	0.39	0.00	0.35	0.36	0.36	0.36	0.00
	2	0.51	0.50	0.55	0.52	0.02	0.84	0.69	0.69	0.74	0.05
	3	0.58	0.56	0.54	0.56	0.01	1.03	0.98	0.98	1.00	0.02
	4	0.36	0.38	0.45	0.40	0.03	3.10	3.21	1.54	2.62	0.54
	5	0.27	0.27	0.26	0.26	0.00	4.92	4.46	4.58	4.65	0.14
	6	0.13	0.15	0.13	0.14	0.01	4.10	4.39	4.17	4.22	0.09
	7	0.07	0.06	0.06	0.07	0.00	4.50	3.61	NA	4.06	0.44
	8	0.06	0.05	0.05	0.05	0.00	2.86	2.54	2.55	2.65	0.11
	9	0.04	0.04	0.04	0.04	0.00	1.67	2.11	1.68	1.82	0.15
	10	0.03	0.03	0.03	0.03	0.00	1.85	1.32	1.75	1.64	0.16
**Gluconic acid [g/L]**	0	NA	NA	NA	NA	NA	NA	NA	NA	NA	NA
	1	0.05	0.05	0.05	0.05	0.00	0.04	0.05	0.04	0.04	0.00
	2	0.92	0.72	0.94	0.86	0.07	0.53	0.28	0.47	0.43	0.08
	3	2.16	1.72	1.79	1.89	0.14	1.05	0.69	0.78	0.84	0.11
	4	5.95	5.78	4.67	5.47	0.40	4.25	4.30	5.25	4.60	0.32
	5	7.91	8.04	8.31	8.08	0.12	7.02	6.11	5.78	6.30	0.37
	6	10.03	9.60	9.79	9.80	0.13	6.07	6.45	6.32	6.28	0.11
	7	11.12	11.18	11.74	11.35	0.20	8.30	6.48	6.66	7.15	0.58
	8	11.54	11.56	12.06	11.72	0.17	9.68	9.16	5.77	8.20	1.22
	9	13.63	12.80	13.59	13.34	0.27	6.54	6.36	9.48	7.46	1.01
	10	14.35	12.45	13.80	13.53	0.56	6.36	6.75	6.46	6.52	0.12
